# LINC01094/miR-577 axis regulates the progression of ovarian cancer

**DOI:** 10.1186/s13048-020-00721-9

**Published:** 2020-10-17

**Authors:** Jing Xu, Ping Zhang, Huajun Sun, Yang Liu

**Affiliations:** 1 Department of Obstetrics and Gynecology, Zibo Hospital of Integrated Traditional Chinese and Western Medicine, Zibo, Shandong Province China; 2 Department of Reproductive Medicine, Linyi People’s Hospital, Fenghuang Street No. 233, Hedong District, Linyi, Shandong Province China; 3 Department of Obstetrics and Gynecology, Yantai Yeda Hospital, Yantai, Shandong Province China; 4 Department of Gynecology, Qingdao Women’s and Children’s Hospital, Qingdao, Shandong Province China

**Keywords:** LINC01094, miR-577, Ovarian cancer, Cancer progression

## Abstract

**Background:**

Long intergenic non-coding RNA 01094 (LINC01094) is probably a novel regulator in cancer biology. This study aimed to probe into the function and mechanism of LINC01094 in ovarian cancer (OC).

**Methods:**

Quantitative real-time polymerase chain reaction (qRT-PCR) assay was utilized to measure LINC01094 and miR-577 expressions in OC tissues and cell lines. Western blot was used to examine the expressions of epithelial-mesenchymal transition (EMT)-related proteins, β-catenin, c-Myc and cyclin D1. Cell counting kit-8 (CCK-8) and Transwell assays were used to detect the proliferation, migration and invasion of SKOV3 and 3AO cells, respectively. Eventually, dual-luciferase reporter gene assay was employed to detect the regulatory relationship between miR-577 and LINC01094.

**Results:**

LINC01094 expression was elevated in OC tissues and cell lines. High LINC01094 expression was associated with higher FIGO stage, lymph node metastasis and the shorter overall survival rate in patients with OC. Meanwhile, LINC01094 knockdown inhibited OC cell proliferation, migration, invasion and EMT. In addition, miR-577 was demonstrated to be a direct downstream target of LINC01094 in OC and inhibition of miR-577 reversed the biological effects of LINC01094 knockdown on OC cells. Additionally, LINC01094 / miR-577 axis regulated the expressions of β-catenin, c-Myc and cyclin D1 in OC cells.

**Conclusion:**

LINC01094 promotes the proliferation, migration, invasion and EMT of OC cells by adsorbing miR-577.

## Introduction

Ovarian cancer (OC) is one of the deadliest cancers among women worldwide, triggering about 151,900 deaths per year [1, 2]. Because of the lack of obvious symptoms at the early stage of the disease, most patients with OC have distant metastasis when being diagnosed, and the five-year survival rate is extremely low [3]. Therefore, it is of great significance to delve into the mechanism of OC tumorigenesis and progression and to find effective markers for early detection and therapeutic targets.

Long non-coding RNA (LncRNA) has been one of the research hotspots in recent years. Composed of more than 200 nucleotides in length, it is a kind of non-coding RNAs (ncRNAs) that cannot encode proteins [4, 5]. Accumulating evidence shows that lncRNAs participate in a variety of biological activities [6]. Importantly, it is found that multiple lncRNAs are abnormally expressed in cancer cells, which modulate carcinogenesis and cancer progression [7]. Exploring the biological functions of lncRNA in the development of OC can provide insights for its diagnosis and treatment. A recent study reports that long intergenic non-coding RNA 01094 (LINC01094) is transcriptionally activated by FOXM1 and it promotes the progression of clear cell renal cell carcinoma [8]. However, in cancer biology, the role of LINC01094 has not been fully elucidated.

MicroRNA (miRNA) is a kind of ncRNA with 20–25 nucleotides. Similar to lncRNA, the abnormal expression of miRNAs is closely interrelated to the development of tumors and features prominently in biological activities, including cell differentiation, stress response, proliferation, apoptosis, migration, and so on [9]. Mounting evidence shows that miRNA can affect the growth and metastasis of OC cells [3, 10, 11]. Previous studies depict that miR-577 expression is abnormally reduced in diverse human malignancies, such as gastric cancer, breast cancer and glioma, suggesting that miR-577 is a tumor suppressor [12–14]. However, the expression and function of miR-577 in OC are not clear.

In this work, we proved that LINC01094 expression was elevated in OC and LINC01094 accelerated cell proliferation, migration, invasion and epithelial-mesenchymal transition (EMT) by targeting miR-577.

## Materials and methods

### Clinical data and ethics statement

Samples of OC tissues and adjacent non-tumor tissues were obtained from 93 patients in the surgery, whose diagnosis were confirmed by histopathology. All experiments were conducted according to the Declaration of Helsinki, and the study was endorsed by the Ethics Committee of the Qingdao Women’s and Children’s Hospital. Moreover, all patients enrolled signed the written informed consent.

### Cell culture

The Institute of Biochemistry and Cell Biology, Chinese Academy of Sciences was the provider of human OC cell lines (SKOV3, HO8910, ES-2, HEY and 3AO cells) and immortalized human ovarian epithelial cell line MOODY. Cells were cultured in Dulbecco’s Modified Eagle’s Medium (DMEM, Gibco, Carlsbad, CA, USA) with 10% fetal bovine serum (FBS; Hyclone, Logan, UT, USA), 100 U/ml penicillin and 100 μg/ml streptomycin (Hyclone, Logan, UT, USA) at 37 °C in 5% CO_2_.

### Cell transfection

Normal control siRNA (si-NC), LINC01094 siRNA, miR-577 mimics and miR-577 inhibitors were obtained from the Genepharma (Shanghai, China). Cell suspensions were prepared with trypsinized SKOV3 and 3AO cells. Then the cells were inoculated in a 6-well plate (1 × 10^6^ cells/well) and cultured. When reaching 80–90% confluence, the OC cells were incubated in fresh medium without serum and antibiotics for 3 h. After that, transfection was performed with Lipofectamine® 2000 (Invitrogen, Carlsbad, CA, USA). After 12 h, the mixture of medium and lipofectamine was replaced by complete medium before the culture was continued for 24 h. After that, the cells were harvested to detect the transfection efficiency and to conduct subsequently experiments.

### Quantitative real-time polymerase chain reaction (qRT-PCR) assay

Based on the manufacturer’s instructions, TRIzol reagent (Takara, Dalian, China) was adopted to extract total RNA from tissues and cells. The cDNA was then synthesized using PrimeScript RT kit (TaKaRa, Dalian, China) and qRT-PCR assay was performed using SYBR Premix Ex Taq II (TaKaRa, Dalian, China) on ABI7500FAST Real-Time PCR system (Applied Biosystems, Waltham, MA, UK), and the data were analyzed by Bio-Rad CFX96TM Manager (Bio-Rad, Hercules, CA, USA). Primers were designed, synthesized and provided by BGI (Shenzhen, China). The relative expressions of LINC01094 and miR-577 were examined with the 2^-ΔΔCt^ method.

### Cell counting kit-8 (CCK-8) assay

CCK-8 kit (Dojindo, Kumamoto, Japan) was employed to evaluate the viability of transfected cells according to the manufacturer’s protocols. Both SKOV3 and 3AO cells transfected with siRNAs were seeded in 96-well plates (1000 cells/well) with 100 μL medium. The cell viability was detected at 0, 24, 48, 72, and 96 h, respectively. At each time point, CCK-8 solution (10 μL/well) was added, and the cells were cultured at 37 °C in 5% CO_2_, and 2 h later, the absorbance of each well was measured employing a Model 680 Microplate Reader (Bio-Rad, Richmond, CA, USA) at a wavelength of 450 nm.

### Transwell assay

In compliance with the manufacturer’s protocol, Transwell chambers with 8 μm pore size (Corning, NY, USA) were utilized to measure cell migration and invasion. Briefly, in cell migration experiments, SKOV3 and 3AO cells (2 × 10^4^ cells) were suspended in 200 μL serum-free medium and placed into the upper chamber of each well, and 600 μL medium containing 10% FBS was dripped into the lower chamber. Cells were cultured at 37 °C for 12 h, and then the migrated cells were fixed with 4% paraformaldehyde and then stained with crystal violet solution. After that, the cells were counted under an inverted microscope. The procedures of invasion experiments were also performed as described above, except that the membrane of the Transwell chambers was coated by Matrigel (30 μg/well; BD, San Jose, CA, USA).

### Luciferase reporter gene assay

The predicted LINC01094 sequence containing miR-577 binding site was amplified and inserted into empty luciferase reporter vector (Promega, Madison, WI, USA) to obtain LINC01094-wild type (WT) reporter vector. Meanwhile, the mutant (MUT) LINC01094 sequence was inserted into empty luciferase reporter vector to obtain LINC01094-MUT reporter vector. LINC01094-WT or LINC01094-MUT was co-transfected into HEK293T cells, respectively, with miR-577 mimic or control miRNA. Forty-eight hours later, relative luciferase activity of each group was measured using dual-luciferase reporter assay system (Promega, Madison, WI, USA).

### Western blot

Total protein was extracted from SKOV3 and 3AO cells with RIPA buffer (Beyotime, Shanghai, China) supplemented with protease inhibitors. The same amount of protein in each group was separated by sodium dodecyl sulfate polyacrylamide gel electrophoresis and then the proteins were transferred to the nitrocellulose (NC) membrane (Pall Life Sciences, Port Washington, NY, USA). After that, the membrane was blocked with 5% skim milk for 1.5 h and then incubated with the corresponding specific primary antibody at 4 °C overnight. Then, the secondary antibody (1:5000) was employed to incubate the membrane at room temperature for 1 h. Ultimately, electrochemical luminescence (ECL) kit (Beyotime, Shanghai, China) was used to detect the immunoreactivity. The primary antibodies used in this work included anti-E-cadherin antibody (Abcam, ab197751, 1:2000), anti-vimentin antibody (Abcam, ab92547, 1:1000), anti-β-catenin antibody (Abcam, ab16051, 1:1000), anti-c-Myc antibody (Abcam, ab32072, 1:2000), anti-cyclin D1 antibody (Abcam, ab16663, 1:2000) and anti-GAPDH antibody (Abcam, ab9484, 1:4000).

### Statistical processing

Statistical analysis was conducted by SPSS22.0 statistical software (SPSS Inc., Chicago, IL, USA). All data were expressed as mean ± standard deviation (SD). The significance of differences between the two groups was examined by *t*-test. GraphPad Prism 8.0 (GraphPad Software Inc., La Jolla, CA, USA) was utilized for drawing the figures. *P*< 0.05 was statistically meaningful.

## Results

### LINC01094 expression was elevated in OC tissues and cell lines

**Fig. 1:**
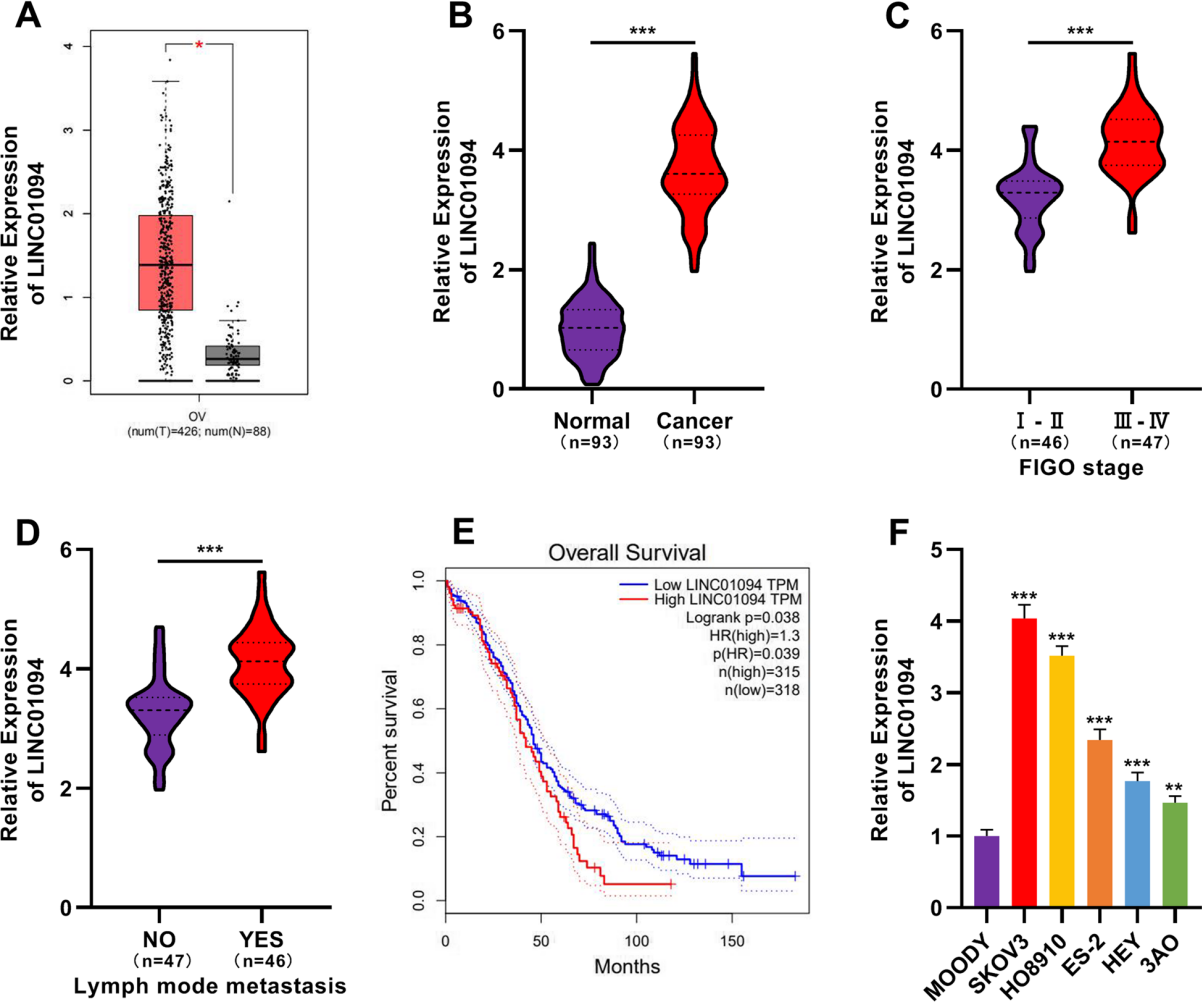
LINC01094 expression was significantly up-regulated in OC tissues and cell lines. **a** Bioinformatics analysis based on GEPIA database showed the expressions of LINC01094 in OC tissues and adjacent normal tissues. **b** The expression of LINC01094 in OC tissues and adjacent tissues was detected by qRT-PCR assay. **c**-**d** The expression of LINC01094 was higher in patients with advanced FIGO stage (**c**) and lymph node metastasis (**d**). **e** GEPIA database was used to perform survival analysis of OC patients with high and low expression of LINC01094. **f** qRT-PCR assay showed that compared with in MOODY cells, the expression of LINC01094 in OC cell lines (SKOV3, HO8910, ES-2, HEY and 3AO cells) was all up-regulated. ** *P* < 0.01 and *** *P* < 0.001

First of all, Gene Expression Profiling Interactive Analysis (GEPIA) database (http://gepia.cancer-pku.cn/help.html) manifested that LINC01094 was differentially expressed in OC tissues and adjacent normal tissues, and LINC01094 expression in cancer tissues was remarkably higher than that in normal tissues (Fig. 1a). Consistently, qRT-PCR assay showed that the expression of LINC01094 in the collected OC samples was markedly higher in comparison with that in adjacent non-tumor tissues (Fig. 1b). To explore the correlation between LINC01094 expression and clinical characteristics, we divided the OC samples into different groups based on FIGO stages and the status of lymphatic metastasis. As shown, the expression of LINC01094 in patients with higher FIGO stage and lymphatic metastasis was markedly higher than that in patients with lower stage and no lymphatic metastasis (Fig. 1c-d). These results demonstrated that LINC01094 was associated with the progression of OC. Importantly, GEPIA database also suggested that the overall survival rate of OC patients with high LINC01094 expression was lower than that in patients with low LINC01094 expression, indicating that LINC01094 had the potential to be a biomarker for evaluating the prognosis the OC patients (Fig. 1e). Next, qRT-PCR assay was conducted to determine the relative expression of LINC01094 in five OC cell lines and immortalized normal ovarian epithelial cell line MOODY. As shown, compared with in MOODY cells, LINC01094 expression in five OC cell lines was significantly up-regulated (Fig. 1f).

### Knockdown of LINC01094 inhibited the proliferation, migration, invasion and EMT of OC cells

**Fig. 2:**
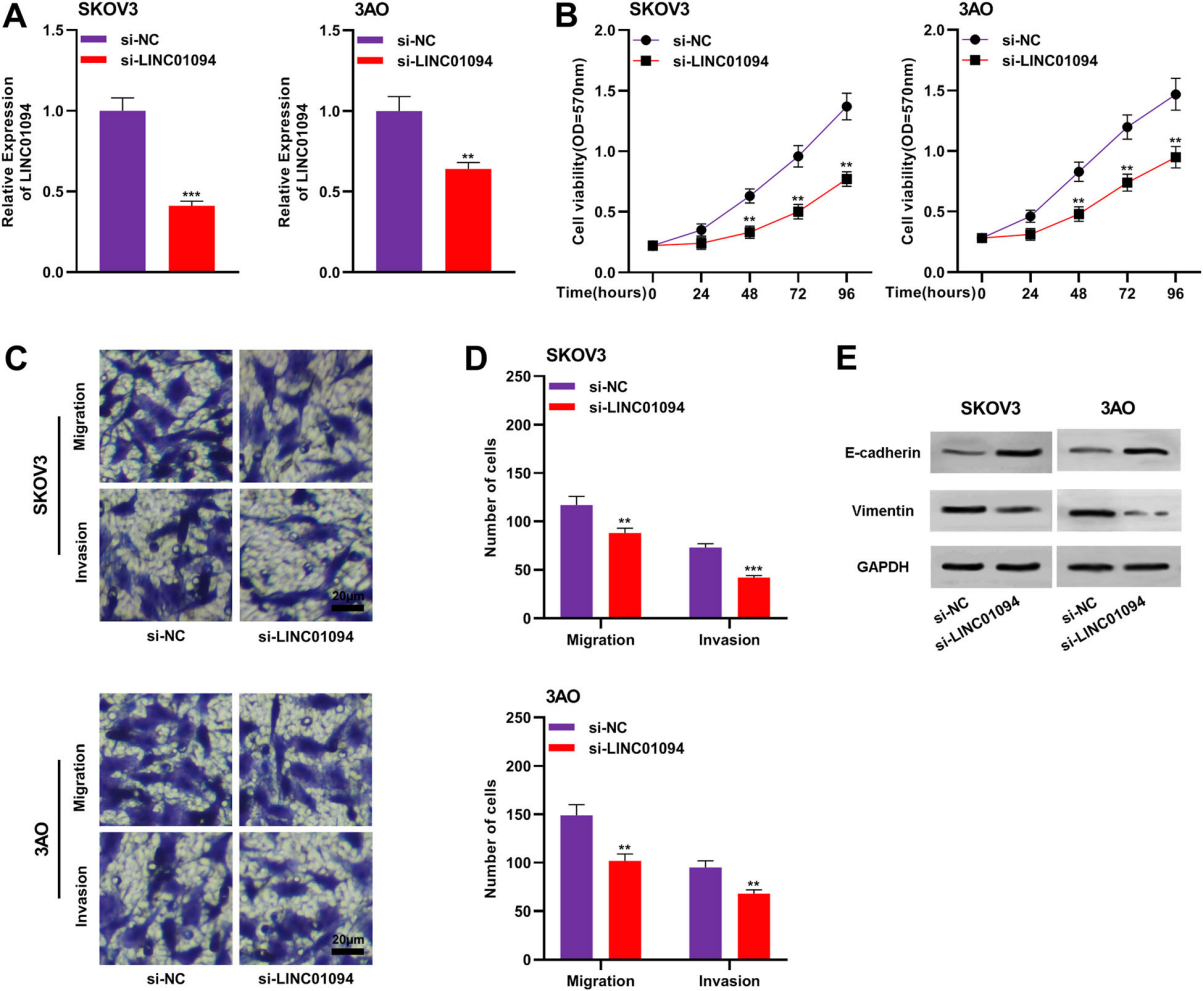
Knockdown of LINC01094 inhibited the proliferation, migration, invasion and EMT of OC cells. **a** SKOV3 and 3AO cells were transfected with si-LINC01094 or si-NC and the inhibition efficiency was detected by qRT-PCR assay. **b** CCK-8 assay was used to detect the effect of LINC01094 on the proliferation of SKOV3 and 3AO cells. **c**-**d** The effects of LINC01094 on the migration and invasion of SKOV3 (**c**) and 3AO (**d**) cells were measured using Transwell assay. **e** Western blot was adopted to analyze the effect of LINC01094 on the expressions of EMT markers E-cadherin and Vimentin. All experiments were performed in triplicate. ** *P* < 0.01 and *** *P* < 0.001

To pinpoint the function of LINC01094 on the malignant behaviors of OC cells, we selected SKOV3 and 3AO cell lines for further exploration. SK-3C and 3AO cells were transfected with si-LINC01094 or si-NC, and the knockdown efficiency was detected by qRT-PCR assay (Fig. 2a). CCK-8 assay results showed that compared to that in the si-NC group, the proliferation of SKOV3 and 3AO cells was significantly inhibited in the si-LINC01094 group (Fig. 2b). Transwell assay demonstrated that knocking down LINC01094 markedly reduced the migration and invasion of SKOV3 and 3AO cells (Fig. 2c-d). In addition, EMT is involved in the metastasis of cancer cells by endowing cancer cells with an invasive phenotype [15]. Therefore, we also explored the impact of LINC01094 on the EMT process. Western blot showed that knocking down LINC01094 could upregulate E-cadherin expression and down-regulate Vimentin expression, which illustrated that LINC01094 regulated the migration and invasion of OC cells partly by modulating EMT (Fig. 2e).

### MiR-577 was a direct downstream target of LINC01094

**Fig. 3:**
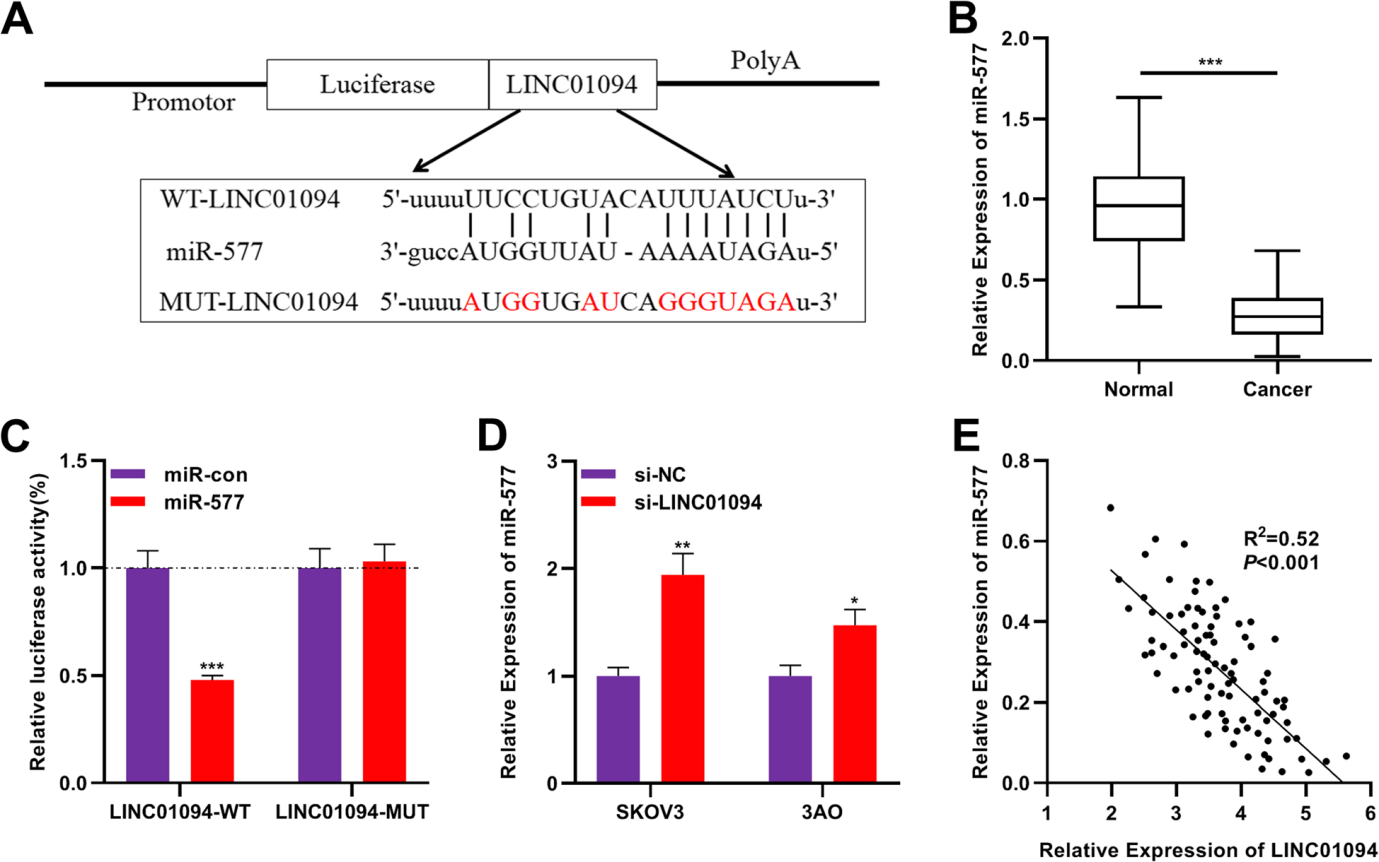
LINC01094 directly targeted miR-577. **a** Bioinformatics analysis was used to predict the binding site between miR-577 and LINC01094. **b** qRT-PCR assay showed that compared with in normal tissues, the expression of miR-577 in OC tissues was significantly down-regulated. **c** The luciferase activity of the cells co-transfected with miR-577-mimics and LINC01094-WT or LINC01094-MUT was determined by dual-luciferase reporter assay. **d** The relative expression of miR-577 in SKOV3 and 3AO cells transfected with si-LINC01094 or si-NC was detected by qRT-PCR assay. **e** Correlation analysis showed the negative correlation between the expressions of LINC01094 and miR-577 in OC tissues. All experiments were performed in triplicate. ** *P* < 0.01 and *** *P* < 0.001

To elaborate on the effect of LINC01094 in OC development, we used the bioinformatics tool StarBase 3.0 (http://starbase.sysu.edu.cn) to predict the target miRNAs potentially regulated by LINC01094 and it was found that miR-577 was a candidate target for LINC01094 (Fig. 3a). To validate this prediction, we determined the expression of miR-577 in OC tissues with qRT-PCR assay, and the results showed that miR-577 expression was remarkably declined in OC tissues (Fig. 3b). Dual-luciferase reporter assay showed that miR-577 mimic observably reduced the luciferase activity of LINC01094-WT reporter, but no significant change could be observed on mutant LINC01094-MUT reporter, indicating that LINC01094 could directly bind to miR-577 (Fig. 3c). In addition, LINC01094 knockdown dramatically enhanced miR-577 expression in SKOV3 and 3AO cells (Fig. 3d). Meanwhile, Pearson’s correlation analysis showed that LINC01094 expression was negatively correlated with miR-577 expression in OC samples (Fig. 3e).

### Inhibition of miR-577 promoted the proliferation, migration and invasion of OC cells

**Fig. 4:**
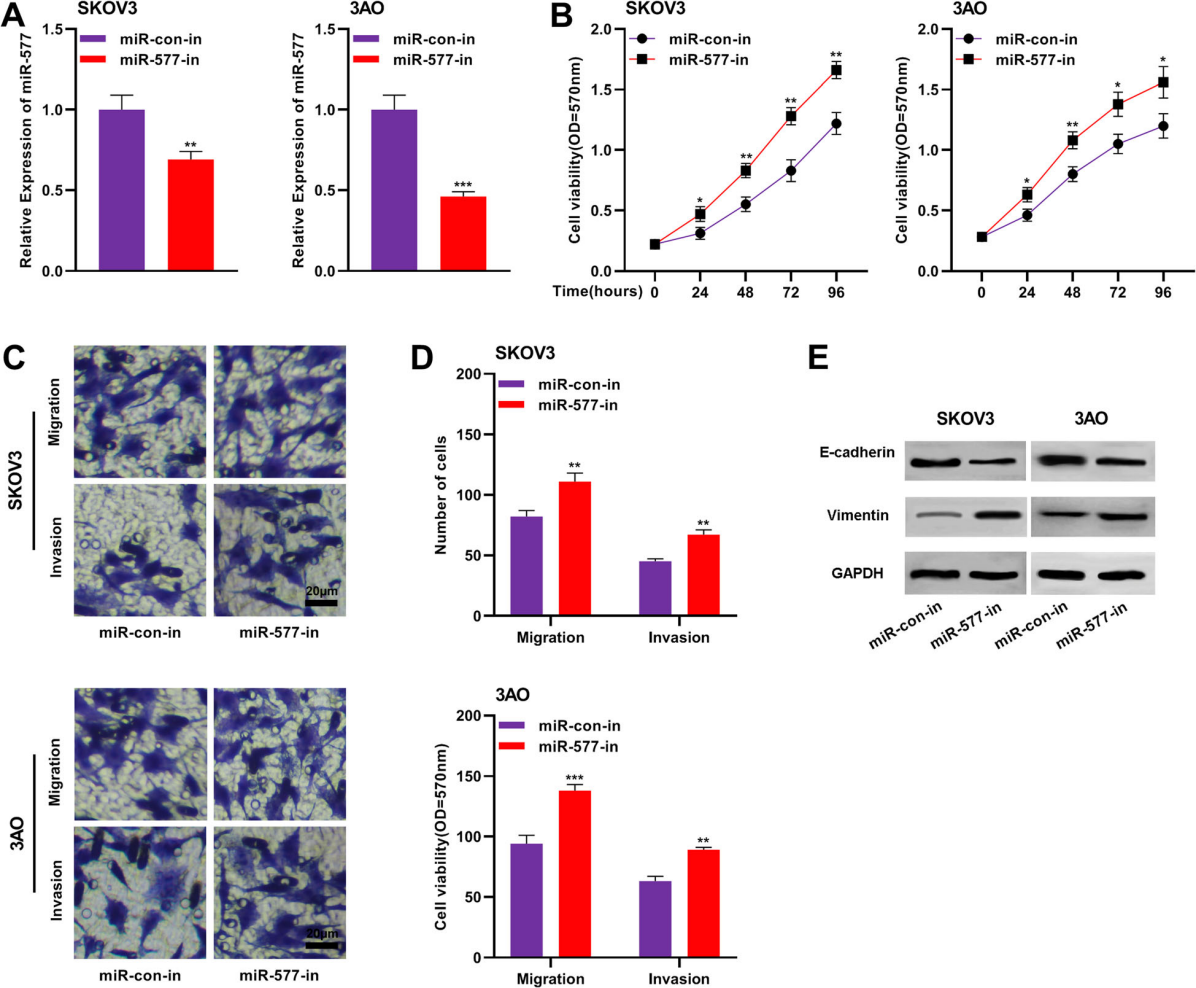
Inhibition of miR-577 promoted the proliferation, migration and invasion of OC cells. **a** SKOV3 and 3AO cells were transfected with miR-577-in or miR-con-in, and the inhibition efficiency was detected by qRT-PCR assay. **b** CCK-8 assay was used to detect the effect of miR-577 on the proliferation of SKOV3 and 3AO cells. **c**-**d** The effects of miR-577 on the migration and invasion of SKOV3 (**c**) and 3AO (**d**) cells were measured using Transwell assay. **e** Western blot was adopted to analyze the effect of miR-577 on the expressions of EMT markers E-cadherin and Vimentin. All experiments were performed in triplicate. ** *P* < 0.01 and *** *P* < 0.001

To fathom the role of miR-577 in regulating the malignant behaviors of OC cells, miR-577 inhibitors or miRNA inhibitor control were transfected into SK-3C and 3AO cells, and the inhibition efficiency was detected by qRT-PCR (Fig. 4a). CCK-8 assay manifested that compared with the control group, inhibition of miR-577 promoted the proliferation of SKOV3 and 3AO cells (Fig. 4b). Transwell analysis also showed that inhibition of miR-577 could significantly promote the migration and invasion of SKOV3 and 3AO cells (Fig. 4c-d). In addition, Western blot results showed that down-regulation of miR-577 expression could decrease the expression of E-cadherin and increase the expression of Vimentin (Fig. 4e). These results suggested that inhibition of miR-577 promoted the proliferation, migration and invasion of OC cells.

### MiR-577 reversed the regulatory functions of LINC01094 on OC cell proliferation, migration and invasion

**Fig. 5:**
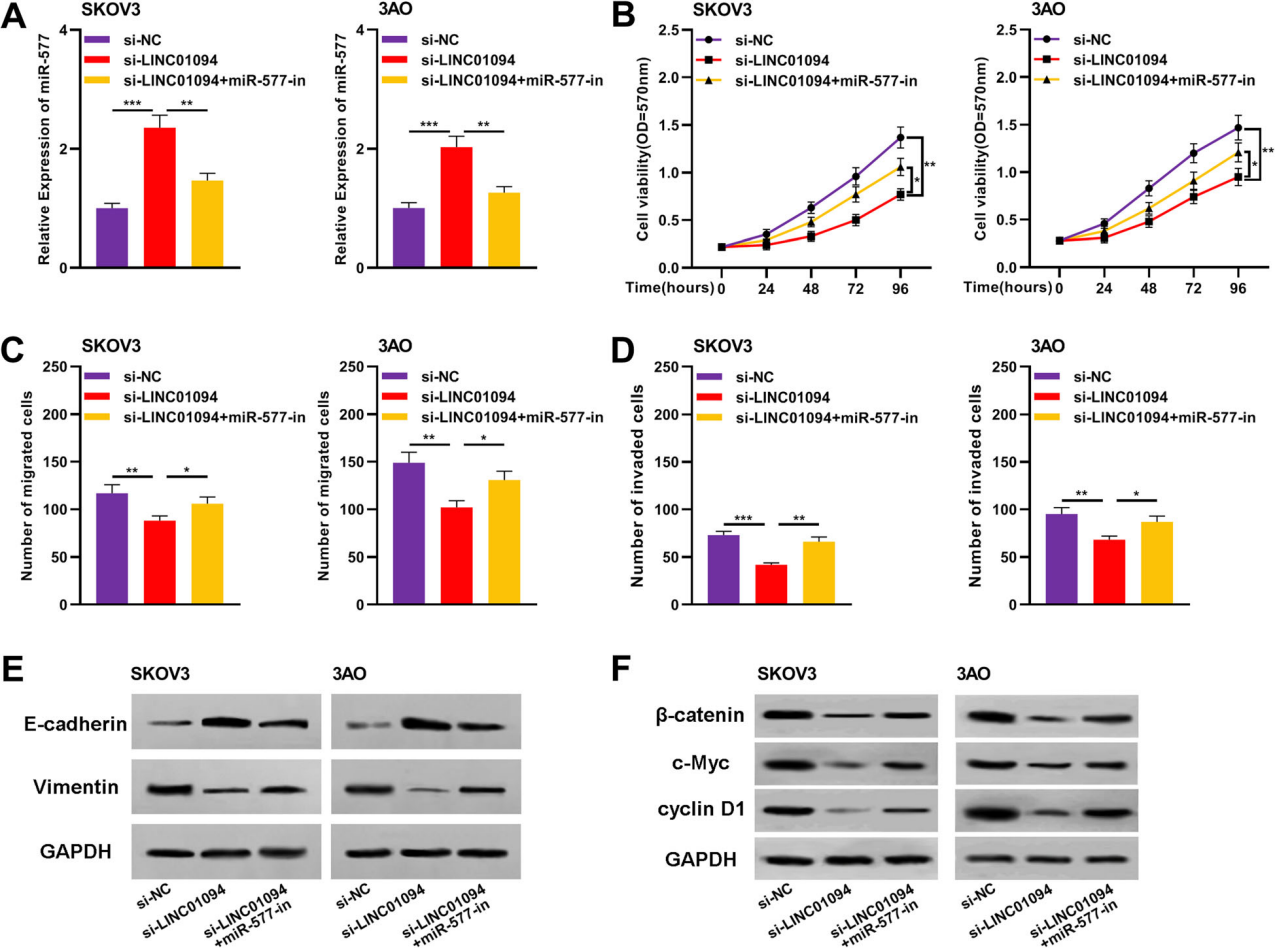
Down-regulation of miR-577 expression partially reversed the biological behavior of OC cells induced by knockdown LINC01094. **a** The expression of miR-577 in SKOV3 and 3AO cells transfected with si-NC, si-LINC01094 or si-LINC01094 + miR-577-in was detected by qRT-PCR assay. **b** CCK-8 assay was used to detect the proliferation of SKOV3 and 3AO cells transfected with si-NC, si-LINC01094 or si-LINC01094 + miR-577-in. **c** Transwell assay was used to detect the migration of SKOV3 and 3AO cells transfected with si-NC, si-LINC01094 or si-LINC01094 + miR-577-in. **d** Transwell assay was used to detect the invasion of SKOV3 and 3AO cells transfected with si-NC, si-LINC01094 or si-LINC01094 + miR-577-in. **e** Western blot was used to analyze the effects of transfection with si-NC, si-LINC01094, or si-LINC01094 + miR-577-in on the expression of EMT markers. **f** Western blot was used to detect β-catenin, cyclin-D1 and c-Myc protein expressions in SKOV3 and 3AO cells transfected with si-NC, si-LINC01094 or si-LINC01094 + miR-577-in. All experiments were performed in triplicate. * *P* < 0.05, ** *P* < 0.01 and *** *P* < 0.001

To investigate whether LINC01094 exerts biological functions through regulating miR-577, we performed a rescue experiment by inhibiting miR-577 expression in SKOV3 cells and 3AO cells with LINC01094 knockdown (Fig. 5a). CCK-8 assay verified that knockdown of LINC01094 inhibited the proliferation of SKOV3 cells and 3AO cells; miR-577 inhibition partially abolished this inhibitory effect (Fig. 5b). Subsequently, Transwell assay demonstrated that knocking down LINC01094 expression remarkably reduced the migration and invasion capacity of SKOV3 cells and 3AO cells; inhibiting miR-577 partially reversed the decrease in the number of migrated and invaded cells (Fig. 5c-d). In addition, Western blot showed that knocking down LINC01094 expression could increase E-cadherin expression and reduce Vimentin expression in SKOV3 and 3AO cells; however, miR-577 inhibitors partially offset the effect of LINC01094 on EMT process (Fig. 5e). Previous studies report that miR-577 exerts its tumor-suppressive function via repressing Wnt/β-catenin signaling [16–18], so we also detected whether LINC01094 could affect the activity of Wnt/β-catenin signaling via regulating miR-577. Western blot was used to detect several Wnt/β-catenin signaling related proteins. It was showed that LINC01094 knockdown reduced β-catenin, cyclin D1 and c-Myc protein expressions in SKOV3 and 3AO cells, while miR-577 inhibitors partially reversed the inhibitory effect of knocking down LINC01094 on these proteins (Fig. 5f). These results indicated that LINC01094 could promote the proliferation, migration, invasion and EMT of OC cells by repressing miR-577 expression and activating Wnt signaling.

## Discussion

It is found that genetic and environmental factors can lead to the occurrence of OC [19]. However, due to the lack of obvious symptoms, effective screening and early diagnosis strategies, exceeding 70% of the patients are diagnosed with advanced OC, resulting in poor prognosis and low survival rate [20]. LncRNA promotes the carcinogenesis, development and metastasis of many human malignancies [21]. Exploring the abnormal expression, function and mechanism of lncRNA in the development of OC can provide biomarkers and therapy targets [22]. For example, a recent study reports that a predictive model, based on the expressions of lncRNAs, GAS5, HCP5, PART1, SNHG11 and SNHG5 included, can identify OC patients at high risk of poor prognosis [23]; the up-regulation of LINC00319 expression accelerates the proliferation, migration and invasion of OC cells [24]; LncRNA PTAR expression is up-regulated in OC and it promotes the EMT and metastasis of OC cells [25]. In this work, we focused on the role of LINC01094 in the progression of OC and found that LINC01094 was highly expressed in OC tissues and cell lines. Additionally, high LINC01094 expression was associated with advanced FIGO stage, lymph node metastasis and poor overall survival rate. Furthermore, in functional assays, we found that LINC01094 knockdown repressed the proliferation, migration, invasion and EMT of OC cells. The above results indicated that LINC01094 probably functioned as a tumor promoter in OC.

The various biological functions of lncRNA are largely dependent on its unique intracellular localization [26]. According to the competitive endogenous RNA (ceRNA) theory proposed in 2011, if the lncRNA is located in cytoplasma and contains miRNA response element (MRE), it probably functions as a molecular sponge to competitively decoy miRNAs, thus modulating the translation of mRNAs [27, 28]. In cancer biology, this mechanism is proved to play a crucial role. For instance, LINC00319 regulates the expression of NACC1 by targeting miR-423-5p to promote the progression of OC [24]; lncRNA HOXD-AS1 facilitates the proliferation and invasion of OC cells by targeting miR-133-3p and activating Wnt / β-catenin signaling pathway [29]. LINC01094 is proved to be a ceRNA for miR-224-5p in clear cell renal cell carcinoma, and it increases the translation of oncogene CHSY1 via competitively binding to miR-224-5p [8]. In the present study, we confirmed that miR-577 was another direct downstream target of LINC01094. It was demonstrated that inhibition of miR-577 promoted the malignant biological behaviors of OC cells. It was also observed that knockdown of LINC01094 increased the expression of miR-577, and LINC01094 expression was negatively correlated with miR-577 expression in OC samples. We also found that the biological functions of LINC01094 was partly dependent on its regulatory function on miR-577. Previous studies demonstrate that miR-577 is a well-recognized tumor suppressor and it negatively regulates a lot of oncogenes and oncogenic pathways, including Sphk2, Smurf1, Rab14, Rab25, LRP6, β-catenin, Wnt2b, and so on [12–14, 16–18, 30–32]. It is possible that LINC01094 can indirectly up-regulate these oncogenes expressions to promote OC progression via repressing miR-577.

The Wnt/β-catenin signaling pathway consists of a series of proteins, which is encoded by proto-oncogenes and tumor suppressors. The interrelationships among these proteins are vital in regulating the process of cell proliferation, apoptosis, differentiation, migration, adhesion, and so on [33]. Activation of the Wnt leads to the stabilization of β-catenin and its accumulation in cytoplasm; β-catenin is then transferred into the nucleus and modulates the transcription of genes [34]. Meanwhile, many oncogenes are transcriptionally activated by Wnt signaling cascades, such as c-myc and cyclin D1 [35]. In OC, Wnt/β-catenin signaling also functions importantly. For instance, PYGB promotes OC cell migration and invasion via modulating the Wnt signaling pathway [36]. By activating Wnt signaling, STAT3 promotes the stemness and spheroid formation of OC cells [37]. Interestingly, many targets of miR-577 are pivotal components of β-catenin/Wnt signaling such as LRP6, β-catenin and Wnt2b, which suggests that miR-577 is a crucial modulator in β-catenin/Wnt signaling. The present work demonstrated that the effects of knocking down LINC01094 on Wnt signaling pathway in OC cells could also be partially reversed by the down-regulation of miR-577. Therefore, these data suggested that LINC01094 activated Wnt signaling pathway through adsorbing miR-577, and promoted the proliferation, migration, invasion and EMT process of OC cells.

In short, in this work, we prove that LINC01094 expression is increased in OC and LINC01094 promotes cell proliferation, migration, invasion and EMT by targeting miR-577 and activating Wnt/β-catenin signaling. This research provides new ideas for OC gene therapy. However, animal experiments are needed to further validate our demonstrations in the following studies. Additionally, other downstream miRNAs of LINC01094 remain to be screened and verified.

## Data Availability

Not applicable.
